# Selective Photophysical Modification on Light-Emitting Polymer Films for Micro- and Nano-Patterning

**DOI:** 10.3390/ma9030121

**Published:** 2016-02-23

**Authors:** Xinping Zhang, Feifei Liu, Hongwei Li

**Affiliations:** Institute of Information Photonics Technology and College of Applied Sciences, Beijing University of Technology, Beijing 100124, China; liufei0307@emails.bjut.edu.cn (F.L.); hw0425@emails.bjut.edu.cn (H.L.)

**Keywords:** light-emitting polymers, selective cross-linking, metalization of phase-separation scheme, plasmonic microstructures

## Abstract

Laser-induced cross-linking in polymeric semiconductors was utilized to achieve micro- and nano-structuring in thin films. Single- and two-photon cross-linking processes led to the reduction in both the refractive index and thickness of the polymer films. The resultant photonic structures combine the features of both relief- and phase-gratings. Selective cross-linking in polymer blend films based on different optical response of different molecular phases enabled “solidification” of the phase-separation scheme, providing a stable template for further photonic structuring. Dielectric and metallic structures are demonstrated for the fabrication methods using cross-linking in polymer films. Selective cross-linking enables direct patterning into polymer films without introducing additional fabrication procedures or additional materials. The diffraction processes of the emission of the patterned polymeric semiconductors may provide enhanced output coupling for light-emitting diodes or distributed feedback for lasers.

## 1. Introduction

Cross-linking between polymer molecules can be achieved chemically, thermally, or photochemically, which have been utilized in the design of light-emitting diodes [1,2]. Cross-linking process has been extensively observed in the interaction between laser beams and polymer molecules [3,4]. The laser-induced cross-linking generally makes the polymeric semiconductor molecules become “inactive”, so that they possess different features from their pristine counterparts, including non-luminescence, enhanced rigidity, insolubility, and high thermal stability. Generally, such effects should be avoided in the optoelectronic devices using the polymeric semiconductor as the active medium [5,6]. However, the laser-induced cross-linking process can be utilized in the micro- or nano-patterning of photonic structures, which are also important for light-emitting diodes. In particular, interference cross-linking has been demonstrated in the fabrication of grating structures on a waveguide with high diffraction efficiency and excellent narrow-band resonance modes [7]. Furthermore, the cross-linking process localized in a small volume induces tensile stress on the surrounding materials that are attached to the polymer film. This has been used to achieve fabrication of metallic photonic structures [8].

In this work, we present a series of experimental studies on the laser-induced cross-linking processes in polymer films, which are applicable in the fabrication of photonic structures. We focus on the selectivity performances of the cross-ling process: (1) spatial selectivity related to high resolution defined by local optical electric field and by the fine-structured templates; (2) molecular phase and light-color selectivity, which is based on the phase separation in the blend film and different optical response of different molecular phases.

## 2. Laser-Induced Cross-Linking in Polymer Film

Irradiation of the polymer film by light within its absorption band will not only induce photo-oxidization of the molecules, but also lead to cross-linking of them. Here we focus only on the later process. Cross-linking of polyfluorene molecules has been described both in the photochemical mechanisms [9] and in the applications in polymeric light emitting diodes [10,11]. Accompanying the cross-linking process, the conjugate polymer degrades quickly and tends to become transparent and nearly non-luminescent. Furthermore, the cross-linked molecules become undissolvable in the organic solvent and exhibit high thermal stability. This implies high stability of the grating structures.

Laser-induced cross-linking results in the reduction in both the thickness and refractive index when a laser beam interacts with a polymer film. This requires that the wavelength of the laser is located within the absorption band of the polymer. Two polymer semiconductors for light-emitting diodes, poly[(9,9-dioctylfluorenyl-2,7-diyl)-*co*-(*N,N0*-diphenyl)-*N,N’*di(*p*-butyl-oxy-pheyl)-1, 4-diamino-benzene)] (PFB) (American Dye Source, Inc., Baie-D’Urfe, QC, Canada) and poly[9,9-dioctylfluorenyl-2,7-diyl)-*co*-1,4-benzo-{2,1’–3}-thiadiazole)] (F8BT) (American Dye Source, Inc., Baie-D’Urfe, QC, Canada), were employed in our experimental studies in this work. Both of these polymers have strong absorption in the UV spectrum. However, blue light at wavelengths longer than 420 nm is only absorbed by F8BT, which facilities selective cross-linking in a blend film. Chemical structures and spectroscopic response of these two polymers have been given in a series of publications [12,13,14,15].

**Figure 1:**
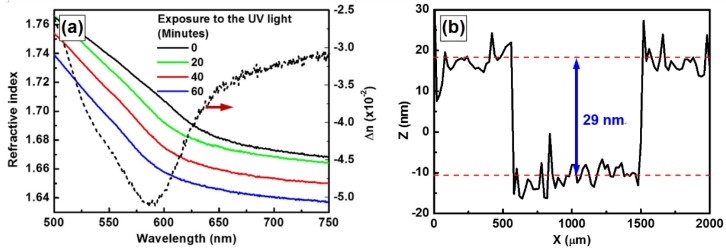
Measurements on (**a**) the refractive index as a function of wavelength and (**b**) the thickness of the PFB film after being irradiated by a UV laser beam at 405 nm for different expose time.

To verify that the laser-induced cross-linking process leads to the reduction in both the thickness and refractive index of the polymer film, we send a continuous-wave (CW) laser beam at 405 nm to a thin film of PFB spin-coated onto a glass substrate with an intensity of 300 mW/cm^2^. In the sample preparation, PFB was first dissolved in chloroform with a concentration of 30 mg/mL. The solution was then spin-coated onto an indium-tin-oxide-coated glass substrate at a speed of 2000 rpm for 30 s. The resultant PFB film has a thickness of about 150 nm. We measured the refractive index of the irradiated PFB film as a function of wavelength using an ellipsometer at different exposure times, as shown in Figure 1a. The solid curves in black, green, red, and blue colors correspond to exposure times of 0, 20, 40 and 60 min. The dashed curve is a calculation by subtracting the black curve from the blue curve, showing the change in refractive index (∆*n*) at different wavelength. The maximum reduction is measured to be about 5 × 10^−2^ at 586 nm. Figure 1b shows measurements on the change in the film thickness of a PFB film within a channel width of about 1 mm after it is exposed to a UV laser beam at 405 nm with an intensity of 500 mW/cm^2^ for about 40 min. A thickness reduction as large as 29 nm can be observed in the data measured using a Dektak profilometer (Veeco Dektak 150, New York, NY, USA). Although Figure 1 shows a relatively rough evaluation, it confirms with solid information that laser-induced cross-linking leads to reduction in both the refractive index and the thickness of a polymer film.

**Figure 2:**
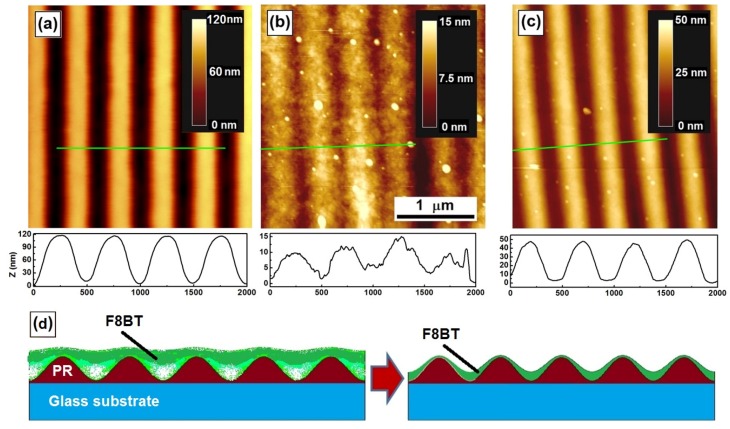
AFM images of: (**a**) the photoresist (PR) grating; and the PR grating spin-coated with F8BT before (**b**) and after (**c**) the laser-induced cross-linking process. (**d**) A schematic illustration of the morphologic modification by laser-induced cross-linking. The lighter colors in the left panel indicate smaller density of F8BT molecules or less filled space before cross-linking.

Laser-induced cross-linking results not only in the reduction of the total volume of the materials, but also in the improvement of the surface smoothness. This can be verified by evaluating the roughness of the PFB film in Figure 1b inside and outside the region that was irradiated by the UV laser beam. The roughness was calculated using the root-mean-square (RMS) value, which were about 3.34 and 3.04 nm for the regions outside and inside the cross-linked regions, respectively. This is more clearly demonstrated by modification on the patterned F8BT thin film, as shown in Figure 2. Figure 2a shows the atomic force microscopic (AFM) image of a photoresist grating before it was spin-coated with F8BT, indicating a modulation depth of about 120 nm. Figure 2b shows the AFM image of the grating after it was spin-coated with F8BT, where a modulation depth of 15 nm and strong roughness can be observed. After the sample was exposed to a laser beam at 405 nm with an intensity of 500 mW/cm^2^ for about 40 min, the modulation depth was increased to 50 nm and the surface smoothness was dramatically improved, as shown in Figure 2c. The plots underneath Figure 2a–c shows the corresponding line scans of the profiles at the marked locations by green lines. The comparison between these plots also demonstrates the improved surface smoothness by the laser-induced cross-linking process. Thus, the F8BT phase has been reduced in the total volume and becomes confined mainly onto the bottom of the grating grooves. Figure 2d described schematically the change in the patterned F8BT film due to the laser-induced cross-linking. Experiments have shown that the spin-coated F8BT could not fill completely the grooves of the photoresist grating [16]. The density of the F8BT molecules reduces from the top to the bottom of the grating grooves. The color change in the left panel of Figure 2d indicates the distribution gradient of the density of the F8BT molecules. The right panel of Figure 2d shows that the cross-linked F8BT sits closely and fits well to the surface of the photoresist grating.

## 3. Spatial Selectivity in Laser-Induced Cross-Linking

### 3.1. Single- and Two-Photon-Induced Cross-Linking Processes

**Figure 3:**
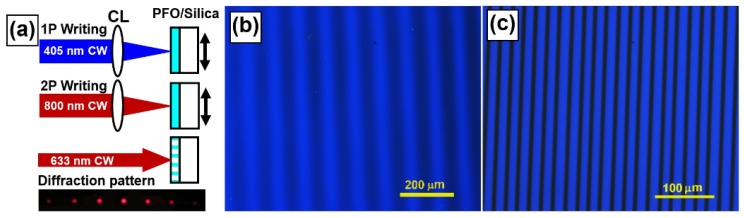
(**a**) Geometry for one-photon (1P) and two-photon (2P) direct writing into a PFB film through focusing a 400-nm CW and an 800-nm femtosecond pulsed laser beam by a cylindrical lens (CL), respectively. High-efficiency diffraction by the 2P-written grating is demonstrated by the diffraction pattern of a red laser at 633 nm. Grating structures written by (**b**) one- and (**c**) two-photon processes.

Focusing a laser beam onto a polymer film is a direct method to achieve microstructures through cross-linking of the molecules. Figure 3a shows the geometry for the direct writing of grating structures using one- (1P) and two-photon (2P) laser-induced cross-linking processes in PFB films. 

In a 1P process, a continuous-wave laser beam at 405 nm from a diode laser was focused by cylindrical lens (CL) onto the PFB film with the substrate mounted on a linear stage and translated manually at fixed steps. The CL has a focal length of about 50 mm and the focusing line at 405 nm has a width of about 50 μm. The laser beam was first expanded before being focused into a line as long as 10 mm. A laser power of about 45 mW and an exposure time of 10 s for the writing of each grating line, which was controlled by a shutter manually. As shown in Figure 3a, the final grating structures have a period of 100 μm. The dark areas correspond to the cross-linked PFB molecules, whereas the bright-blue-colored areas correspond to the slightly or non-cross-linked PFB molecules. It is obvious that we did not get grating lines with high-contrast edges. This is because there is a Gaussian-like distribution of the intensity along the profile of the laser beam and cross-linking process may be also initiated by the weak or even scattered UV light. This limits the spatial resolution of the written grating structures.

In contrast, the 2P process enabled much improvement in the fabrication through cross-linking of the PFB molecules, as shown in Figure 3b by the optical microscopic images of the PFB grating. In the 2P fabrication, femtosecond laser pulses from a Ti:sapphire amplifier at 800 nm with a pulse length of about 150 fs, a repetition rate of 1 kHz, and a pulse energy of about 100 μJ were focused by a CL with a focal length of 50 mm. Again, the sampled was mounted on a linear stage and translated at fixed steps manually during the writing processes. Each exposure of a grating line takes 10 s. This 2P interaction allows much improved spatial resolution and a grating period smaller than 20 µm. The cross-linked areas, as shown by the high-contrast dark lines in Figure 3b are narrower than 6 µm with very clear edges. The threshold effect in the two-photon process is crucial for such high-contrast writing of grating structures. Such a grating exhibits high efficiency of diffraction. The bottom panel of Figure 3a shows the diffraction pattern of red laser beam at 633 nm when it was sent to the grating structures shown in Figure 3c. Bright laser spots can be observed at different orders of diffraction.

### 3.2. Interference Cross-Linking with High Spatial Resolution

**Figure 4:**
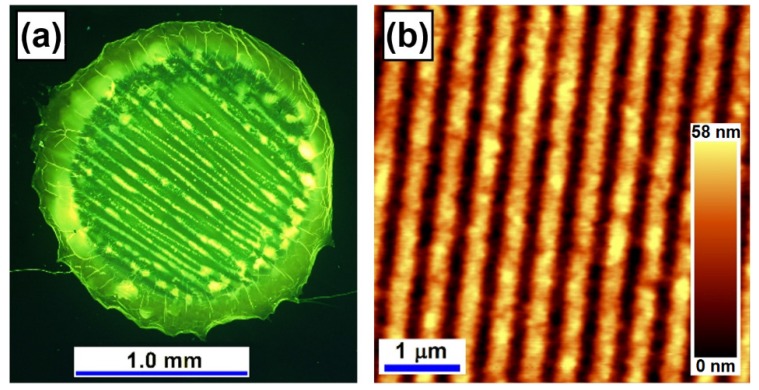
(**a**) Optical microscopic image of the cross-linked F8BT film after the liftoff process by rinsing the sample with chloroform. (**b**) The AFM image of the grating structures produced by direct interference cross-linking and subsequent liftoff process.

Cross-linking processes can be confined into very small volumes, implying high spatial resolution. Experiments indicated a sub-100-nm scale resolution with possibility for further improvements. Therefore, interference cross-linking has been demonstrated for the fabrication of photonic structures with excellent optical response [7]. Figure 4 shows experimental results of grating fabrication using interference crosslinking in a F8BT thin film. A He-Cd laser at 325 nm was employed in a two-beam interference scheme with a separation angle of about 41°, so that a relief grating with a period of about 470 nm was obtained. After the grating was rinsed in chloroform, the non-cross-linked F8BT was removed and the grating structures composed of cross-linked F8BT molecules remained on the substrate, as shown in Figure 4a. Thus, the modulation depth of the grating was increased and a maximum groove depth of less than 60 nm was obtained, as shown in Figure 4b. Such a grating has shown excellent optical and thermal stability [7], implying possible practical applications. Such a fabrication method is also strongly dependent on the exposure time. Since the cross-linked F8BT molecules become insoluble and rinsing in chloroform can be considered as a development process, the quality of the resultant grating structures is determined and can be optimized by the exposure dose.

## 4. Phase-Selective Laser-Induced Cross-Linking in Polymer Blends for Plasmonic Patterning

Solution-processed gold nanoparticles enabled development of a variety of fabrication techniques for plasmonic nanostructures [17,18]. Advantages of these methods involve simplicity, large-area fabrication, and flexible tunability in spectroscopic response of the finished structures, as has been demonstrated in References [17,18]. However, fabrication of large-area plasmonic structures with optical response in the infrared spectral range beyond 1 μm is still a challenge for this method. Annealing of the spin-coated films of colloidal gold nanoparticles generally produces structures in nanoscale, so that the resultant plasmon resonance spectrum is located in the visible spectrum. Alternatively, filling the grooves of grating structures is flexible in tuning the size of the gold structures, where the period of the template photonic structures can be adjusted, for example, in the stage of interference lithography. In this case, the spectrum of plasmon resonance is limited in bandwidth due to the homogeneity of the dimensions of the gold nanostructures defined by the periodic master grating. Therefore, a large-area template is expected to provide microstructures randomly distributed in a large range in the scale of micrometers.

### 4.1. Selective Cross-Linking of the Phase-Separation Scheme of a Polymer-Blend Film

**Figure 5:**
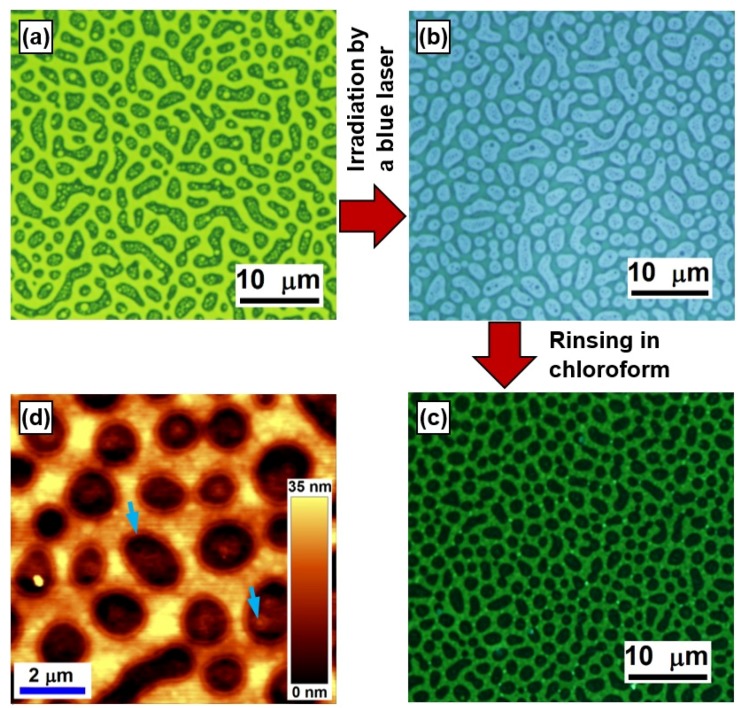
Optical microscopic image of the phase-separation scheme of the F8BT:PFB blend film: before (**a**) and after (**b**) blend film is exposed to a blue laser beam at 457 nm for about 20 min; and (**c**) after the sample is rinsed in chloroform. (**d**) AFM image of the structures shown in (**c**).

In the preparation of the blend film, PFB and F8BT were dissolved in xylene with a concentration of 15 mg/mL before they were mixed together with a volume ratio of PFB:F8BT = 1:2. The mixed solution was spin-coated onto a glass substrate at a speed of 3000 rpm. Figure 5a shows the optical microscopic image of the fresh blend film under UV excitation. The bright yellow domains correspond to the F8BT-rich phase, whereas the blue-greenish domains circled by the yellow correspond to the PFB-rich phase. However, yellow particles can also be observed in the PFB-rich domains, corresponding to the small F8BT phases embedded in the PFB.

A blue laser at 457 nm was sent to the blend film with an intensity of about 100 mW/cm^2^. Since PFB has nearly no absorption at 457 nm, only F8BT molecules were excited by the blue laser beam and cross-linking process took place only for the F8BT molecules. Thus, a cross-linked “skeleton” formed by the cross-linked F8BT molecules after the blend film is exposed to the blue laser for about 60 min. As a result, F8BT emission was hardly observed after the above exposure process. As shown in Figure 5b, the blend film emits blue light under UV excitation, implying nearly pure PFB emission and non-luminescent F8BT-phase domains observed under the optical microscope. 

The blend film is then immersed and rinsed in chloroform. The cross-linked F8BT is not dissolved in chloroform and stays firmly on the substrate, while the PFB-rich phase was removed completely by the rinsing process. Thus, only the “skeleton” network of cross-linked F8BT molecules was kept on the glass substrate, as shown by fluorescent optical microscopic image in Figure 5c. Although the F8BT molecules became nearly non-luminescent after being cross-linked, weak emission in green still enabled clear observed of the F8BT-bordered network under UV excitation. The black holes correspond to the original PFB-rich phases that have been removed by the chloroform-rinsing process. Figure 5d shows the AFM image of the structures in Figure 5c. A modulation depth of about 35 nm was observed with a domain size ranging from sub-1 μm to microns. Due to heterojunctions formed at the interface between the F8BT- and PFB-rich phases, ring structures can be observed at the outer edge of each domain. These ring structures can be used to evaluate the width of the heterointerfaces [19]. Furthermore, small ring structures can also be observed in the “holes”, corresponding to the small yellow-greenish particles in Figure 5a, which result from the small fractions of F8BT-rich phase embedded in the PFB. These finer structures inside each hole provide mechanisms for the formation of porous gold grains in the subsequent metallization processes.

### 4.2. Metallization of the Selectively Cross-Linked Phase-Separation Pattern

**Figure 6:**
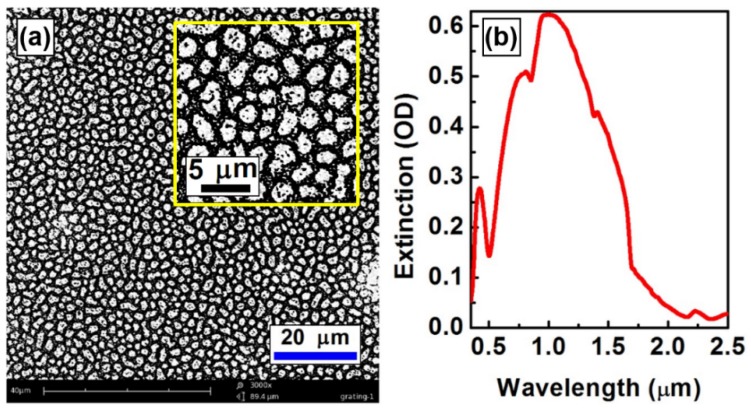
(**a**) SEM image of the metalized phase-separation scheme of the F8BT:PFB blend film; (**b**) Optical extinction spectrum measured on the structures shown in (**a**).

Solution-processed gold nanoparticles were first spin-coated onto the phase-separation scheme shown in Figure 5c,d in the metallization process. The gold nanoparticles were synthesized chemically and were suspended in xylene with a concentration of 100 mg/mL, where the diameter of the gold nanoparticles is distributed in a range from 5 to 10 nm [17,18]. Spatially modulated by the patterns in Figure 5c,d, the colloidal solution was confined mainly into the holes formed by the cross-linked F8BT borders. A subsequent annealing process at about 350 °C enabled melting of the gold nanoparticles and further confinement of the gold into the holes of the phase-separation scheme, forming randomly distributed gold grains as large as microns, as shown in the scanning electron microscopic (SEM) image in Figure 6a. The cross-linked F8BT phase stays stably on the substrate without being destroyed by the heating process. Thus, the stable network of the cross-linked polymer functions as a mask and enabled formation of gold grains as a reversed image of the mask in Figure 5c. Two features may be observed with the gold nanostructures: (1) Bordered by the cross-linked F8BT phase, the gold was confined into irregularly shaped grains in micron scales, corresponding to the holes shown in Figure 5c,d; (2) The cross-linked F8BT phase in the PFB-rich phase lead to the porous structures inside each gold grain, as illustrated more clearly in the inset of Figure 6a.

Figure 6b shows optical extinction spectrum measured on the metalized structures consisting of porous gold micro-grains in Figure 6a. Strong optical extinction can be observed in a broad spectral range from 500 nm to nearly 2 μm. According to a rough evaluation using Figure 6a, although the size of gold grains is distributed in a range from sub-1 μm to sub-5 μm, the small holes separate the large gold grains into domains with an average dimension of about 1 μm. This explains why the plasmon resonance of the metalized structures is peaked at 997 nm with amplitude of 0.62 OD (optical density), as shown by the optical extinction spectrum in Figure 6b. Such structures are potentially important for exploring optical or optoelectronic devices operating in the infrared.

## 5. Conclusions

We investigated different selective interactions between thin films of polymeric semiconductors and different laser radiations. Selective cross-linking induced multifold photo-physical and microscopic modifications, enabling exploration of new mechanisms for the fabrication photonic structures. In particular, although the cross-linked F8BT molecules have lost their conjugation performance and become non-luminescent, the spatial selectivity and high spatial resolution of the uncross-linked molecules still keep their original semiconductor performance. Thus, the resultant patterned polymers can be used to produce active photonic devices or structured light-emitting diodes with designed output coupling performance. This is also important for exploring optically or electrically pumped laser devices.
